# Phase-Based and Lifetime Health System Costs of Care for Patients Diagnosed with Leukemia and Lymphoma: A Population-Based Descriptive Study

**DOI:** 10.3390/curroncol31080313

**Published:** 2024-07-25

**Authors:** Anubhav Agarwal, Natasha Kekre, Harold Atkins, Haris Imsirovic, Brian Hutton, Doug Coyle, Kednapa Thavorn

**Affiliations:** 1 School of Epidemiology and Public Health, University of Ottawa, Ottawa, ON K1G 5Z3, Canada; 2 Ottawa Hospital Research Institute, Ottawa, ON K1H 8L6, Canada; 3 Department of Medicine, The Ottawa Hospital, Ottawa, ON K1H 8L6, Canada; 4 Royal Ottawa Mental Health Center, Ottawa, ON K1Z 7K4, Canada

**Keywords:** leukemia, lymphoma, Ontario, health system costs, lifetime costs, phase-based costing, joinpoint modelling

## Abstract

Hematologic cancers, notably leukemias and lymphomas, pose significant challenges to healthcare systems globally, due to rising incidence rates and increasing costs. This study aimed to estimate the phase and lifetime health system total costs (not net costs) of care for patients diagnosed with leukemia and lymphoma in Ontario, Canada. We conducted a population-based study of patients diagnosed between 2005 and 2019, using data from the Ontario Cancer Registry linked with health administrative databases. Costs were estimated using a phase-based approach and stratified by care phase and cancer subtype. Acute lymphocytic leukemia (ALL) patients had the highest mean monthly initial (CAD 19,519) and terminal (CAD 41,901) costs among all cancer subtypes, while acute myeloid leukemia (AML) patients had the highest mean monthly cost (CAD 7185) during the continuing phase. Overall lifetime costs were highest for ALL patients (CAD 778,795), followed by AML patients (CAD 478,516). Comparatively, patients diagnosed with Hodgkin lymphoma (CAD 268,184) and non-Hodgkin lymphoma (CAD 321,834) had lower lifetime costs. Major cost drivers included inpatient care, emergency department visits, same-day surgeries, ambulatory services, and specialized cancer drugs. Since 2005, the cost structure has evolved with rising proportions of interventional drug costs. Additionally, costs were higher among males and younger age groups. Understanding these costs can help guide initiatives to control healthcare spending and improve cancer care quality.

## 1. Introduction

According to the Canadian Cancer Society (CCS), blood cancers account for about 10% of all new cancer cases and about 3% of all cancer deaths in Canada [1]. Its incidence in Canada has been increasing over the past few decades. In 2023, it was estimated that there would be approximately 18,400 new cases of leukemia and lymphoma cancers in Canada and an estimated 6300 deaths from these cancers. Non-Hodgkin lymphoma alone accounts for 10,900 new cases and 3100 deaths out of those totals, and 6400 new cases and 3100 deaths out of those were estimated to be of leukemias [1].

Although the number of new cases of cancer is increasing every year, Canada has made remarkable progress in the field of cancer prevention, detection, and treatment since the early 1990s. High-quality clinical and translational research has made significant advances in the treatment of cancer, leading to an increase in the survival rates of patients living with cancer. According to CCS, the overall 5-year survival rate for blood cancers is now over 60%. The largest increases in 5-year survival between 1992 and 2017 were for chronic myeloid leukemia (25% points) and acute lymphocytic leukemia (23% points), followed by non-Hodgkin lymphoma (21% points), which are largely attributed to improvements in treatments [1,2].

The total health system cost of cancer care in Canada was estimated to be CAD 18.4 billion in 2021 [3]. This includes costs related to treatment, supportive care, and end-of-life care. These costs are expected to continue to increase as the population ages and cancer rates continue to rise. The costs are particularly higher for blood cancers than for other types of cancer [4]. Oliveira et al., (2016) estimated the mean lifetime (25-year) net costs of care for lymphoma to be CAD 107,514 for adult females, CAD 114,574 for adult males, and for leukemia to be CAD 138,749 among adult females and CAD 128,641 among adult males [5]. It is important to note that leukemias and lymphomas are heterogeneous groups of cancers, and thus, the cost of cancer care can vary widely depending on the cancer subtype, phase of care, available treatment options, and other factors; therefore, it is necessary to have a more nuanced look at the costs of these cancers. Further, with the recent clinical advancements in leukemia and lymphoma care and the introduction of expensive therapies, it is critical to have up-to-date and accurate cost estimates related to their treatment. As financial pressures mount on publicly funded healthcare systems like Canada’s, precise future planning and prioritization become essential. This requires reliable estimates of treatment costs, which are crucial for assessing the economic value and allocating resources for cancer care. Our study aimed to address this gap by estimating the phase-specific and lifetime health system total costs of care for patients diagnosed with each leukemia and lymphoma subtype, the drivers of these costs, and the temporal cost trends in the province of Ontario, Canada.

## 2. Materials and Methods

We performed a population-based, descriptive study to estimate the costs incurred by patients diagnosed with leukemia and lymphoma as their primary cancer, from the public payer perspective. This study was conducted within ICES (formerly known as the Institute of Clinical Evaluative Sciences), a prescribed entity under section 45 of Ontario’s Personal Health Information Protection Act and did not require a separate research and ethics board review.

### 2.1. Study Participants

**Figure 1:**
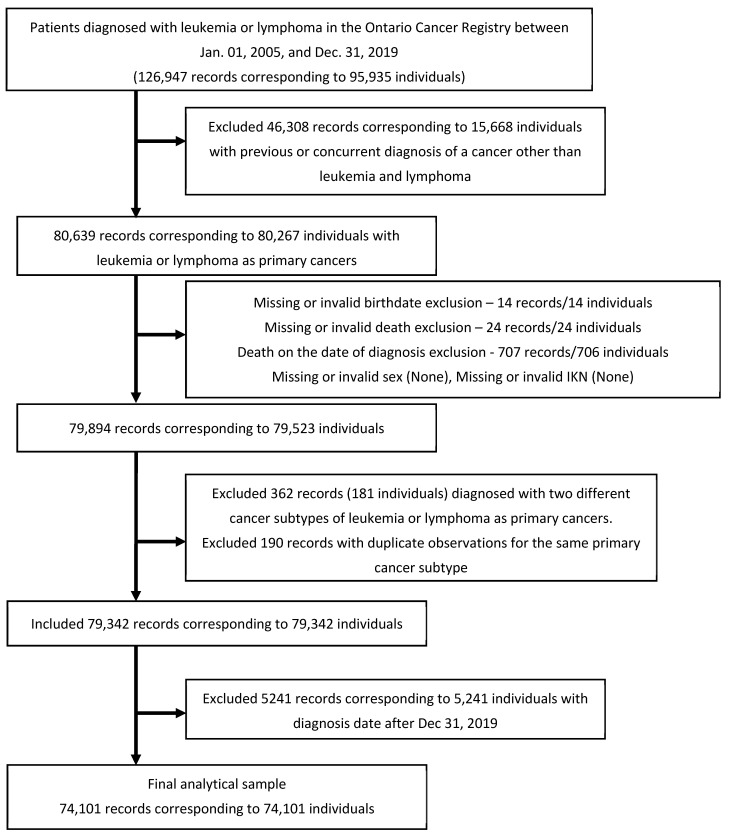
Cohort creation flow diagram.

All patients, of all ages, diagnosed with leukemia or lymphoma as primary cancer between 1 January 2005 and 31 December 2019, who had at least one contact with the health system in Ontario were included in the study. Patients were identified from the Ontario Cancer Registry (OCR) using the relevant International Classification of Diseases for Oncology, 3rd Edition/World Health Organization 2008 (ICD-O-3/WHO 2008) definitions (Table S1) [6]. Ontario non-residents, patients who were not continuously Ontario Health Insurance Plan (OHIP) eligible from 1 year prior to the index (diagnosis) event date and at least 1 year after the index date or until death, patients with missing or invalid information on sex, date of birth, IKN (unique encoded identifier to link ICES databases), date of diagnosis of cancer, or ICD-O-3 code of cancer at the time of diagnosis, or whose cancer diagnosis was made only on the death certificate were excluded from this study. (To be eligible for OHIP coverage, a person must have Canadian citizenship or an approved immigration status, make Ontario their primary place of residence, and be physically present in Ontario for at least 153 days within a 12-month period. Source: https://www.ontario.ca/document/resources-for-physicians/registration-ontario-health-insurance-coverage#section-1; accessed on 15 July 2024.) Figure 1 shows the flow diagram of cohort creation with relevant details. Based on the ICD-O-3/WHO 2008 classification codes, leukemias were further classified into the following cancer subtypes: acute lymphocytic leukemia (ALL), chronic lymphocytic leukemia (CLL), acute myeloid leukemia (AML), and chronic myeloid leukemia (CML). Leukemia patients that were not classified into the above subtypes were classified into the “Other leukemias” category. Lymphoma patients were classified into two subtypes—non-Hodgkin lymphoma (NHL) and Hodgkin lymphoma (HL). 

### 2.2. Data Sources

Individual records for leukemia and lymphoma patients from the OCR were linked to several other health administrative databases (Table S2). These datasets were linked using unique encoded identifiers and analyzed at ICES. Inpatient hospitalization data were derived from the Canadian Institute for Health Information Discharge Abstract Database. The Ontario Health Insurance Plan claims database provided information on physician care in Ontario. Information on the cost of administered drugs was obtained from the New Drug Funding Plan (NDFP) and Ontario Drug Benefit (ODB) claims. ODB is unavailable for most individuals under 65 years, which limits our ability to account for outpatient drug information for this age group. The information about radiation and systemic therapy was obtained from the Cancer Activity Level Reporting. Costs for same-day procedures, emergency visits, and ambulatory care were obtained from the National Ambulatory Care Reporting System. Other databases used included the Ontario Case Costing Initiative, GAPP Decision Support Systems (physician payments), ICES Physician Database, drugs list, the Postal Code Conversion File, the Reference Files (look-up tables), information about Ontario healthcare institutions, the estimated schedule of benefit prices associated with each OHIP fee code and suffix, the home care database, the Ontario home care administrative system, the national rehabilitation reporting system, the Ontario mental health reporting system, and the same-day surgery database. 

The care costs were categorized into the following components:

1. Inpatient costs: Includes hospitalization-related costs.
2. Outpatient costs: Includes general outpatient clinic costs.
3. Physician services cost: Includes physician fees (including outpatient visits), shadow billings, and capitation payments.
4. Prescription drug costs: Includes the costs of drugs covered under the ODB programme. ODB covers prescription drugs for patients meeting certain criteria including, age (65+ years), income, and residency status.
5. Specialized cancer drug costs: Includes the costs of drugs that are covered under NDFP and includes intravenous systemic chemotherapy drugs.
6. Emergency department, same-day surgery, and ambulatory care costs: refer to the costs incurred from same-day surgery visits, emergency department visits, and high-cost ambulatory care visits to the dialysis clinics and cancer clinics. This does not include physician billings incurred during any of the abovementioned visits.
7. Home and community care costs: Includes the costs incurred from home care services, rehabilitation, complex and continuing care, and long-term care costs.
8. Other costs: Includes laboratory billings, non-physician billings, and assisted device costs.

### 2.3. Cost Estimation

We estimated the cost of care from the perspective of the publicly funded healthcare system and included all costs borne by the Ontario government. We used a hybrid costing methodology developed at ICES (GETCOST SAS macro) to compute total health system costs from administrative databases for a given timeframe [7,8]. The method uses both top-down and bottom-up costing methodologies depending on the nature of the cost. The top-down approach divides total healthcare spending from a comprehensive healthcare budget by sector and provider. This is suitable, for instance, when unit prices are unknown for short-term episodes such as for inpatient hospitalizations, and ambulatory care (includes same-day surgeries, emergency department visits, cancer clinic visits, and dialysis clinic visits). In such cases, the resource intensity weights of the short-term episode are multiplied by the annual cost per weighted case. For long-term episodes such as institutional care, costs per weighted day are calculated. In contrast, a bottom-up approach calculates costs by starting with the healthcare usage and expenses at the individual level, which are then summed up. The costs of individual encounters such as laboratory tests and outpatient physician visits are calculated using this bottom-up approach. All costs were converted to 2023 Canadian dollars (CAD) using Statistics Canada’s Consumer Price Index for healthcare (Table S3) [9].

### 2.4. Statistical Analysis

We used the phase-based approach to estimate the lifetime costs of cancer care. Prior research has shown that healthcare costs associated with cancer care have three distinct phases of cost accumulation that follow a U-shaped pattern [10,11]. Firstly, the initial phase—a high-cost accumulation phase following the diagnosis—is marked by intense resource use resulting from treatment initiation including, chemotherapy, radiation, and hospitalizations. Secondly, the continuing phase—a comparatively lower cost-intensity phase—involves monitoring, detecting relapses, and managing complications arising from the treatments received during the initial phase. Lastly, the terminal phase—a high-cost accumulation phase—encompasses inpatient care including palliative and end-of-life care [12]. 

We applied the Joinpoint Regression Model (JRM) method to segment the phases of cancer care [13]. This involved a piecewise linear regression analysis using the National Cancer Institute’s Joinpoint Trend Analysis Software to identify statistically significant shifts in the trend in monthly healthcare costs [14]. The analysis focused on monthly all-cause healthcare costs as the dependent variable, with time (in months) serving as the independent variable. We developed two distinct models: one for the initial phase following cancer diagnosis and another for the terminal phase leading up to death, both employing a homoscedastic, uncorrelated error model. To find the best fit for our data, we utilized a grid search, a sequential algorithm-based method. Joinpoints, or significant changes in the cost trend, were determined using a permutation test (with 5000 permutations at a 0.05 significance level). Confidence intervals for the true average percentage change were then calculated using the empirical quantile method. This approach, leveraging the full dataset, allowed us to accurately delineate the cost dynamics for different phases of cancer care.

Based on the results of JRM (Figure S1a,b), the following definitions of phase durations were identified:

- The initial phase: the first 9 months immediately after diagnosis;
- The terminal phase: 3 months before death;
- The continuing phase: time between the initial and terminal phase.

For patients surviving less than or equal to 3 months, all time was allocated to the terminal phase. Patients were considered to have entered the initial phase only if they survived for more than 3 months. For patients surviving between 3 and 12 months, the last 3 months of life (before death) were allocated to the terminal phase and the remaining time to the initial phase. Patients must survive more than 12 months before time accounting in the continuing phase can begin—the first 9 months after diagnosis in the initial phase; the last 3 months before death in the terminal phase; and the remaining time in the continuing phase. Thus, depending on the length of the survival period from the time of diagnosis, not all patients contributed costs to all phases. Based on earlier research, the prediagnosis phase was defined as the last three months just before diagnosis [5,15]. The same phase definitions were used for all cancers to ensure comparability among cancers [5]. We defined a month to comprise a 30-day duration. 

The phase-based modelling approach was used to account for censoring. Individuals are censored when they do not experience the event of interest, i.e., death, within the study’s observation period. This would lead to incomplete costs as full healthcare expenditures would not be available for all individuals in the study because some of those individuals will die outside the study window [16]. 

The estimation of lifetime costs involved the following steps. Firstly, the observation time and costs for each patient were allocated to the specific phases following the phase definitions as outlined above. Mean monthly all-cause healthcare costs per phase were estimated for each of the subtypes of leukemia and lymphoma [16,17,18]. In the next step, survival probabilities for each 30-day time interval from the time of diagnosis to the end of follow-up were calculated using the Kaplan–Meier (KM) method. Finally, the estimated mean phase-specific monthly costs and survival estimates were combined to compute lifetime costs using the below formula:

$$Lifetime\;costs=\sum_{i=1}^{T}S\left(i\right)Cp$$

where *S*(*i*) represents the survival probability at the *i*th 30-day period since the index time, and *C_p_* is the mean monthly phase-specific cost. *C_p_* can take one of the three values depending on whether the *i*th time interval was allocated to the initial, or the continuing, or the terminal phase for the patient. *T* represents the end of the follow-up.

We calculated means, medians, and 95 % confidence intervals for the continuous variables, and estimated frequencies and percentages for categorical variables. We present the overall lifetime costs as well as by age and sex. The subgroup analysis was pre-specified and exploratory in nature, and no formal hypothesis testing was performed. Statistical analyses were performed using SAS Enterprise Guide 7.1 [19].

## 3. Results

### 3.1. Patient Characteristics

Our cohort included 47,255 lymphoma patients and 26,846 leukemia patients with 64 years and 66 years as the respective median age at diagnosis (Table 1). Males comprised 54.6% of lymphoma patients and 57.9% of leukemia patients. Around 89% of the lymphoma patients were diagnosed with non-Hodgkin lymphoma. Among leukemias, 40.3% of the patients were diagnosed with chronic lymphocytic leukemia, followed by acute myeloid leukemia (24.4%). Other leukemias (12.5%) represented a heterogeneous mix of ‘left-over’ leukemia types, which poses challenges in interpretation and limits our ability to draw specific conclusions from these data. The median follow-up time from the date of diagnosis for lymphoma patients was 1603 days, and for leukemia patients, it was 1327 days.

### 3.2. Phase-Specific Mean Monthly Costs

**Figure 2:**
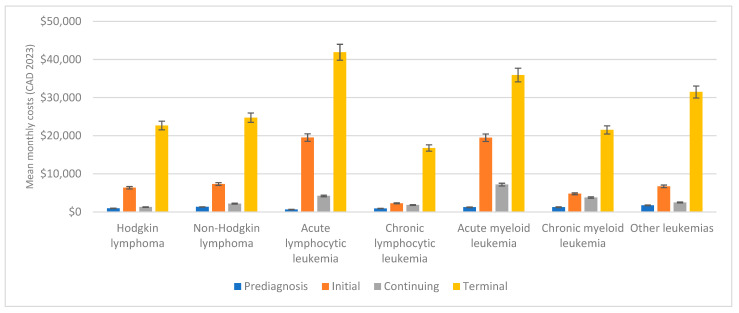
Phase-specific mean monthly costs by cancer subtype (with bars for 95% confidence intervals) (CAD, 2023).

Figure 2 illustrates the phase-specific mean monthly all-cause healthcare costs associated with different cancer subtypes.

#### 3.2.1. Prediagnosis Phase

The mean monthly costs in the prediagnosis phase were the lowest compared to other phases of care, across all cancer subtypes. This phase represented the 3-month period before the cancer diagnosis was made and had costs associated with preliminary tests, diagnostics, and consultations. The costs ranged from CAD 655 (95% CI: 602–708) for ALL patients to CAD 1730 (95% CI: 1621–1839) for patients diagnosed with other leukemias. 

#### 3.2.2. Initial Phase

The initial phase, which lasted up to 9 months after diagnosis, saw a significant increase in the mean monthly costs for all cancer subtypes. ALL patients had the highest initial phase mean monthly costs at CAD 19,519 (95% CI: 19,120–19,918), followed by AML patients at CAD 19,474 (95% CI: 19,081–19,867). The lowest initial phase costs were for CLL patients at CAD 2270 (95% CI: 2176–2363).

#### 3.2.3. Continuing Phase

During the continuing phase, which is the time between the initial and the terminal phases, costs were relatively low. This phase encompassed the continuous treatment, monitoring, and management of the disease, potentially involving chemotherapy, radiation, or other therapies. Additionally, as costs were distributed over an extended period, the monthly expenses for this phase were generally lower. HL patients had the lowest continuing phase mean monthly costs of CAD 1286 (95% CI: 1199–1373), while AML patients had the highest mean monthly costs in the continuing phase at CAD 7185 (95% CI: 6851–7519).

#### 3.2.4. Terminal Phase

The terminal phase, defined as the last 3 months before death, is characterized by the highest costs among all phases, which is consistent across all cancer subtypes. This phase involves more intensive and costly care mostly due to increased inpatient care, medical interventions, or palliative care. ALL patients had the highest terminal phase mean monthly costs at CAD 41,901 (95% CI: 39,678–44,124), while CLL patients had the lowest terminal phase mean monthly costs at CAD 16,790 (95% CI: 16,200–17,380).

#### 3.2.5. Trends in Phase-Specific Mean Monthly Costs

A notable trend was observed in the phase-specific mean monthly costs since 2005 (Figure S2a–g). Specifically, when examining lymphoma patients, the prediagnosis costs have remained relatively stable between 2005 and 2019. While continuing costs have shown a slight increase, this trend has been more pronounced in recent years. For lymphoma patients, there was a steady increase in initial costs, and a steep rise was observed in terminal costs, which surged by 30% between 2005 and 2007 and between 2017 and 2019 for HL patients, and by 37% for NHL patients during the same period. 

A similar yet more pronounced trend was identified in the case of leukemias, particularly for ALL and AML patients, for which initial, continuing, and terminal costs all had a steep rise between 2005 and 2019. Most remarkable was the rise in the costs during the initial phase of care for both ALL and AML patients, which is distinctly different from patients diagnosed with other cancer subtypes. The terminal care costs have also soared during the study period for all leukemia patients. 

The analysis of phase-specific mean monthly costs also included examining different age groups (age at diagnosis). For all cancer subtypes except CLL, which predominantly affects adults, the highest initial and terminal costs were observed in the age group of 0 to 17 years old, gradually decreasing with each subsequent age group. The prediagnosis and continuing care costs were higher in older age groups than their younger counterparts (Figure S3a–g).

### 3.3. Component Contribution in Phase-Specific Costs

**Figure 3:**
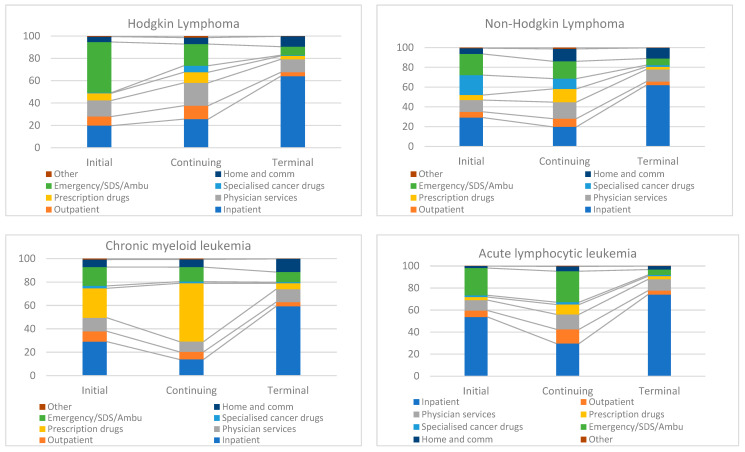

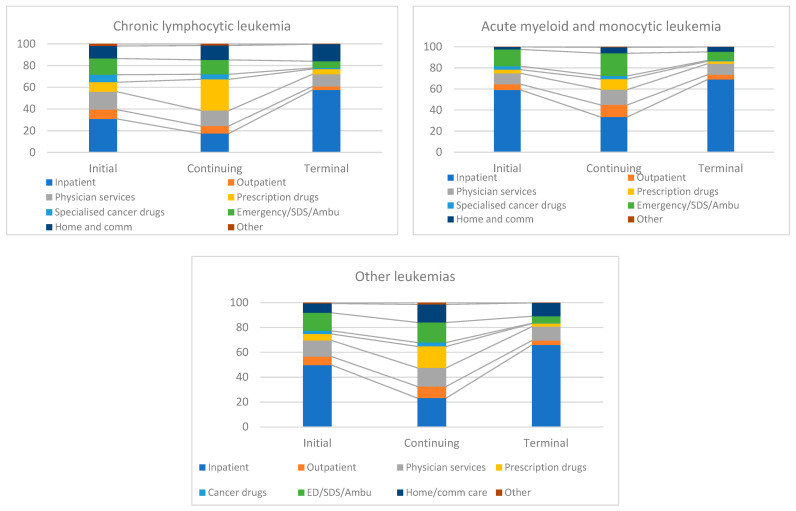
Overall component distribution in phase-specific costs for lymphomas and leukemias (%). Note: ED—emergency department; SDS—same-day surgery; Ambu—ambulatory care; Home/comm—home and community care.

Figure 3 presents the component distribution in each of the three phases.

For HL patients, emergency, same-day surgery, and ambulatory care services represent a significant portion of the costs in the initial phase at 45.4%, followed by inpatient costs (19.7%) and physician services (14.4%). The continuing phase had a more even distribution of costs, with inpatient services at 25.6%, physician services at 20.4%, and emergency care services at 19.4%. In the terminal phase, the proportion of inpatient costs rises significantly to 64.1%. Home and community care accounted for 9.3% of the costs in the terminal phase of HL patients. Among patients diagnosed with NHL, specialized cancer drugs contributed significantly to the initial and continuing phases at 20.1% and 10.4%, respectively. Inpatient costs remain a major driver in all three phases of care for NHL patients.

In patients diagnosed with leukemias, for both ALL and AML, inpatient cost was the major component in all three phases of care followed by emergency, same-day surgery, and ambulatory care services. The cost components and their distribution were similar for CLL and CML patients. Inpatient services were a major cost contributor in the initial and terminal phases; however, unlike patients with acute leukemias, prescription drugs (which primarily included ODB drugs for 65+ patients) were also a major cost driver in the continuing phase for both the chronic leukemia patients—28.7% for CLL and 49.9% for CML. In CLL patients, home and community care were also a significant contributor at 11.5%, 13.2%, and 15.6% in the initial, continuing, and terminal phases of care, respectively. A similar composition was observed in patients with other leukemias, with inpatient costs accounting for 49.7% of the costs in the initial phase and 66.0% in the terminal phase. Prescription drugs were the second major cost driver after inpatient costs in the continuing phase of care for patients diagnosed with other leukemias.

The category “Other costs”, contributed from 0.2% to 2% for different types of cancers, and included assisted device costs, lab costs under OHIP, and non-physician fees. 

We analyzed temporal trends for the proportions of cost components in the three phases of care from 2005 to 2019 for all cancer subtypes combined, which are presented in Figure S4a–c. Over the years, for the initial phase, the proportion of inpatient care costs has decreased from 43% in 2005–2007 to 35.6% in 2017–2019, while the proportion of ambulatory, emergency and same-day surgery, and specialized cancer drugs has increased from 15% to 25.6% and 11.3% to 12.8%, respectively, between 2005–2007 and 2017–2019, indicating a shift in the cost structure of care for patients diagnosed with cancer during the initial phase. Notable changes were observed in the cost component distribution in the continuing phase with a considerable increase in the proportions of specialized cancer drugs, which has more than doubled between 2005 and 2019 from 4.5% to 9.8%. The cost proportion of emergency, same-day surgery, and ambulatory care services also increased from 15.7% to 22.4% in the continuing phase over the study period. Further, the inpatient cost proportion decreased slightly during this phase between 2005 and 2016 from 21.4% to 20.2% but has seen a slight rise since then to 21.7% in 2017–2019. Physician services cost proportion also saw a decline from 16.7% in 2005–2007 to 14% in 2017–2019 in the continuing phase of care. Not much change was observed in the cost components and their distribution in the terminal phase of care, with a slight increase in the proportion of emergency, same-day surgery, and ambulatory services costs (from 6.3% in 2005–2007 to 7.9% in 2017–2019), and physician services costs (10.4% in 2005–2007 to 13.2% in 2017–2019). Additionally, there was a slight decline (from 11.2% in 2005–2007 to 7.3% in 2017–2019) in the home and community care costs proportion during the terminal phase of cancer care.

### 3.4. Lifetime Costs

Table 2 shows the lifetime health system costs for patients diagnosed with leukemia and lymphoma. The table also shows the time spent in each phase and the total cost by phase per patient. The overall estimates of lifetime costs per patient varied widely, from a high of CAD 778,795 (95% CI: 737,045–820,544) for ALL patients to a low of CAD 262,583 (95% CI: 254,794–270,372) for CLL patients. AML patients had a lifetime cost of CAD 478,516 (95% CI: 460,411–496,620) and the corresponding value for CML patients was CAD 444,867 (95% CI: 422,484–467,251). The lifetime costs of the patients diagnosed with two subtypes of lymphomas were relatively lower, at CAD 268,184 (95% CI: 252,681–283,687) for HL patients and CAD 321,834 (95% CI: 316,159–327,510) for NHL patients. A consistent pattern can be seen across all cancer subtypes whereby a large share of lifetime cost is formed of total costs incurred during the continuing phase. This is because of the much longer time that the patients spent in the continuing phase as compared to the initial or the terminal phases (even though mean monthly costs in the continuing phase are typically lower than the initial or the terminal phase). When stratifying the analysis by age, individuals aged 65 years and above had the least variability in estimates of lifetime costs compared to estimates across other age groups for the various cancer subtypes (Table S4c–e). Among children aged 0–17 years, lifetime cost was highest for the AML (CAD 1,234,258; 95% CI: 998,413–1,470,103). And in the 18–64 age group, ALL had the highest lifetime cost (CAD 954,372; 95% CI: 866,771–1,041,974). When stratified by sex, the lifetime costs for males were slightly higher than females for all cancer subtypes except for AML. However, even with lower costs in all three phases of care, females spent more time in the continuing phase than males (Table S4a,b). 

## 4. Discussion

Our study provides the latest and most comprehensive cost estimates associated with care for patients diagnosed with leukemias and lymphomas in the publicly funded healthcare system of Ontario. Using real-world data, we show that health systems costs vary by cancer subtypes. Inpatient, emergency services, and drugs drive the costs for most of these cancers in all three phases of care. We also found that the costs have risen over time and vary by both sex and age at diagnosis. Additionally, there are age-related variations in care costs, and the cost structure has evolved with the increased use of intensive drug therapies over time. 

Our study adopted the phase-based approach, which has become a widely used method for estimating lifetime costs in the presence of heavy censoring [5,10,16,18,20,21]. Our results are consistent with those of other studies with costs exhibiting a U-shaped pattern, with the highest mean monthly costs being incurred during the initial and terminal phases and relatively low costs during the continuing phase of care for all cancer subtypes [5,21,22]. Inpatient services, emergency service, ambulatory care, same-day surgery, and drugs (both specialized cancer drugs and other prescription drugs) were the main cost drivers in providing care for patients diagnosed with leukemias and lymphomas. 

We found noticeably higher mean monthly costs for ALL patients and AML patients than other types of leukemias and lymphomas, during the initial and terminal phases, which could be because patients diagnosed with these subtypes of cancers often require hospitalization soon after diagnosis for first-line chemotherapy, which is usually not the case in other cancer subtypes. Additionally, as compared to other leukemias, ALL and AML patients require high-cost systemic therapies that must be administered in a hospital setting to allow for close monitoring [4].

We also observed a shift in the costs after the patients were diagnosed with cancer for the three phases of care, as depicted in Figure S4. These trends are multifaceted and reflect changes in healthcare practises, patient management, and the development of new treatments over the years in Canada. The decrease in the proportion of inpatient care costs suggests a trend towards shorter hospital stays or more efficient inpatient care, possibly due to improvements in surgical techniques, early discharge programmes, or a shift towards outpatient management where feasible [23]. 

Conversely, the significant increase in the proportion of the costs of prescription drugs and specialized cancer drugs, especially during the continuing phase, indicates a growing reliance on high-cost pharmaceutical interventions, such as targeted therapies and immunotherapies for cancer treatment [23,24]. This shift has important implications for healthcare financing, patient access to care, and the overall sustainability of cancer treatment, particularly as the prevalence of cancer continues to rise in Canada and globally. 

Our study shows that ALL patients incurred the highest lifetime costs. When stratified by age (see Table S4), AML patients had the highest lifetime cost among children aged 0–17 years, and ALL patients had the highest lifetime cost among adults aged 18–64. Patients diagnosed with these leukemia subtypes (ALL or AML) exhibit high rates of inpatient and emergency care services, which are cost intensive. The Milliman Research Report on the cost burden of blood cancer care in the USA, commissioned by the Lymphoma & Leukemia Society, has also reported that the highest costs are borne by acute leukemia patients due to huge costs billed by inpatient hospitals, which was six times the costs incurred by patients with other blood cancers [4].

Further, sex disparities in lifetime costs were evident, with males exhibiting slightly higher costs than females, except in the case of AML. This contrast may be attributed to factors such as older age at diagnosis and the superior survival rates observed among females [25,26,27,28]. Our analysis concurred that, on average, females tended to be diagnosed at an older age, except for AML patients. Consistent with prior research, lifetime costs decreased with age, potentially due to a treatment paradigm emphasizing palliative care over intensive therapies for older patients and because older patients spent relatively less amount of time in the continuing phase than their younger counterparts [28]. 

Our study has several strengths. We employed an objective data-driven methodology (joinpoint regression modelling) to identify the durations of phases of care that consider the shape of cost trajectories specific to our study population [29]. In contrast to most United States-based studies, which mostly included patients 65 years and older, our study included patients of all age groups [21,30]. We estimated detailed costs for patients diagnosed with subtypes of leukemia and lymphoma, which have not been previously reported for Ontario. This study’s estimates and findings may be relevant to similar settings where such detailed cost estimates are not available for patients diagnosed with leukemia and lymphoma subtypes. 

However, our study has certain limitations. The primary limitation of this study is the lack of a control group, which restricts our analysis to estimate the total healthcare costs without assessing net or attributable costs of cancer care. Additionally, the ICES costing methodology does not fully account for radiation and certain systemic chemotherapies, as well as out-of-pocket expenditures, likely resulting in an underestimation of the actual costs. In Ontario, we only had data on outpatient drugs covered by the publicly funded ODB programme, which mainly serves those aged 65 and older, limiting our ability to capture the drug-related costs borne by private insurance or patients. We also could not estimate costs by cancer stage, as this information was unavailable from the OCR for the study period. Additionally, the OCR does not capture information on cancer relapse, which could have highlighted cost variations between patients with new diagnoses (de novo) and those with relapsed conditions, particularly during the continuing phase of care. Recently, novel methods have been developed to determine relapse using electronic health records, which could be adapted to determine cost differences between de novo and relapsed patient groups [31]. Lastly, although previous studies have demonstrated the accuracy and reliability of OCR, which captures around 95% of all cancer diagnoses in Ontario, using ICD codes from large administrative databases may introduce potential coding errors that could impact the accuracy of disease identification and categorization [32,33,34].

## 5. Conclusions

This study provides a descriptive analysis of healthcare expenditures across different phases of care as well as lifetime costs for patients diagnosed with leukemia and lymphoma. Our findings indicate substantial variability in costs, driven primarily by inpatient and emergency care services. This study highlights sex disparities in costs, with males generally incurring higher expenses except in acute myeloid leukemia patients. This study acts as a foundation for future research focusing on estimating net costs for each of the subtypes of leukemia and lymphoma. This study also offers valuable insights into the financial burden of patients diagnosed with leukemia or lymphoma from a health system perspective and can aid in the development of cost-effective treatment strategies.

## Figures and Tables

**Table 1:** Patient characteristics (*N* = 74,101).

Characteristic	Lymphoma		Leukemia	
	Number	Percent	Number	Percent
Overall sample	47,255	100	26,846	100
Type of cancer				
Hodgkin lymphoma	5233	11.1		
Non-Hodgkin lymphoma	42,022	88.9		
Acute lymphocytic leukemia			2694	10.0
Chronic lymphocytic leukemia			10,830	40.3
Acute myeloid leukemia			6553	24.4
Chronic myeloid leukemia			3405	12.7
Other leukemias			3364	12.5
Median age at diagnosis in years (Q1, Q3)	64.0 (51.0, 75.0)		66.0 (53.0, 77.0)	
Sex				
Male	25,805	54.6	15,531	57.9
Female	21,450	45.4	11,315	42.1
Neighbourhood income quintile				
1—Low	8682	18.5	5242	19.6
2	9456	20.1	5394	20.2
3	9224	19.6	5199	19.4
4	9491	20.2	5421	20.2
5—High	10,188	21.7	5497	20.6
Rural residence	5916	12.5	3614	13.5
Died during follow-up	18,759	39.7	12,916	48.1
Median follow-up time in days (Q1, Q3)	47,255	1603.0 (582.0, 3061.0)	26,846	1327.5 (383.0, 2837.0)
Year of diagnosis				
2005	2424	5.1	1480	5.5
2006	2461	5.2	1526	5.7
2007	2612	5.5	1683	6.3
2008	2633	5.6	1651	6.1
2009	2608	5.5	1701	6.3
2010	2983	6.3	1946	7.2
2011	3019	6.4	1793	6.7
2012	3286	7.0	1740	6.5
2013	3440	7.3	1888	7.0
2014	3447	7.3	1934	7.2
2015	3405	7.2	1961	7.3
2016	3736	7.9	1813	6.8
2017	3741	7.9	1921	7.2
2018	3694	7.8	1909	7.1
2019	3766	8.0	1900	7.1

Notes: Q1 = 25th percentile; Q3 = 75th percentile.

**Table 2:** Time spent in each phase, total cost by phase, and lifetime costs per patient (all ages).

Cancer Subtype	n	Number of Months in Phase			Cost by Phase			Lifetime Cost
		Initial	Continuing	Terminal	Initial	Continuing	Terminal	
Hodgkin lymphoma	5233	8.66 (8.62–8.70)	84.49 (82.98–85.99)	2.72 (2.68–2.77)	53,832 (52,632–55,033)	198,747 (185,322–212,172)	15,604 (14,727–16,481)	268,184 (252,681–283,687)
Non-Hodgkin lymphoma	42,022	8.42 (8.40–8.44)	67.54 (67.02–68.07)	2.57 (2.56–2.58)	55,233 (54,613–55,853)	226,450 (221,942–230,958)	40,151 (39,604–40,699)	321,834 (316,159–327,510)
Acute lymphocytic leukemia	2694	8.6 (8.54–8.67)	80.06 (77.84–82.27)	2.51 (2.44–2.58)	157,173 (153,959–160,386)	588,158 (551,397–624,918)	33,464 (31,689–35,240)	778,795 (737,045–820,544)
Chronic lymphocytic leukemia	10,830	8.74 (8.71–8.76)	71.43 (70.49–72.37)	2.80 (2.78–2.82)	19,075 (18,291–19,860)	214,484 (208,500–220,469)	29,023 (28,003–30,043)	262,583 (254,794–270,372)
Acute myeloid leukemia	6553	7.34 (7.26–7.43)	47.13 (45.41–48.86)	2.27 (2.24–2.30)	98,752 (96,759–100,745)	314,499 (299,884–329,115)	65,264 (63,768–66,761)	478,516 (460,411–496,620)
Chronic myeloid leukemia	3405	8.4 (8.33–8.46)	64.17 (62.28–66.05)	2.69 (2.65–2.73)	37,317 (35,588–39,046)	371,618 (352,728–390,508)	35,932 (34,168–37,697)	444,867 (422,484–467,251)
Other leukemia	3364	8.29 (8.21–8.37)	65.41 (63.35–67.48)	2.04 (1.98–2.09)	41,867 (39,474–44,260)	207,872 (191,328–224,416)	43,189 (41,336–45,042)	292,929 (272,139–313,719)

Costs in CAD 2023; 95% confidence intervals in parentheses.

## Data Availability

The dataset from this study is held securely in coded form at ICES. While legal data sharing agreements between ICES and data providers (e.g., healthcare organizations and government) prohibit ICES from making the dataset publicly available, access may be granted to those who meet pre-specified criteria for confidential access, available at www.ices.on.ca/DAS (email: das@ices.on.ca).

## Supplementary Materials

The following supporting information can be downloaded at: https://www.mdpi.com/article/10.3390/curroncol31080313/s1, Table S1. List of inclusionary ICD-O-3/WHO 2008 codes. Table S2. Database sources. Table S3. Historic CPI for health and personal care in Canada. Figure S1. (a,b): Joinpoint modelling results to determine the duration of (a) initial phase and (b) terminal phase. Figure S2. (a–g) Trends in phase-specific mean monthly costs grouped by year of diagnosis for subtypes of leukemia and lymphoma (3-year groupings) (CAD 2023). Figure S3. (a–g): Phase-specific mean monthly costs grouped by age at diagnosis (CAD, 2023). Figure S4. Temporal trends in cost component contribution (% of total costs) for all cancer types combined—(a) initial phase, (b) continuing phase, (c) terminal phase. Note: SDS—same-day surgery; Ambu—ambulatory care; Home and comm—home and community care. Table S4. (a–e): Lifetime costs per patient, stratified by sex and age at diagnosis.
