# Supplementary material for: Phase-Based and Lifetime Health System Costs of Care for Patients Diagnosed with Leukemia and Lymphoma: A Population-Based Descriptive Study
Source: Curr Oncol. 2024 Jul 25;31(8):4192–208. doi: 10.3390/curroncol31080313 (PMC11352321; doi:10.3390/curroncol31080313)
Supplement: Supplementary file 1 [file curroncol-31-00313-s001.zip › curroncol-3068478-supplementary.pdf]

Supplementary Tables and Figures

Table S1. List of inclusionary ICD-O-3/WHO 2008 codes.

Site Group	ICD-O-3 Site	ICD-O-3 Histology (Type)
**Lymphomas**		
**Hodgkin lymphoma**		
Hodgkin—Nodal	C024, C098–C099, C111, C142, C379, C422, C770–C779	9650–9667
Hodgkin—Extranodal	All other sites	
**Non-Hodgkin Lymphoma (NHL)**		
NHL—Nodal	C024, C098, C099, C111, C142, C379,C422, C770–C779	9590–9597, 9670–9671, 9673, 9675, 9678–9680, 9684, 9687–9691, 9695, 9698–9702, 9705, 9708–9709, 9712, 9714–9719, 9724–9729, 9735, 9737–9738, 9811–9818, 9823, 9827, 9837
NHL—Extranodal	All sites except C024, C098–C099, C111, C142, C379, C422, C770–C779	9590–9597, 9670–9671, 9673, 9675, 9678–9680, 9684, 9687, 9688, 9689–9691, 9695, 9698–9702, 9705, 9708–9709, 9712, 9714–9719, 9724–9729, 9735, 9737, 9738
	All sites except C024, C098–C099, C111, C142, C379, C420-C422, C424, C770–C779	9811–9819, 9823, 9827, 9837
**Leukemias**		
**Lymphocytic Leukemia**		
Acute Lymphocytic Leukemia		9826, 9835–9836
	C420, C421, C424	9811–9819, 9837
Chronic Lymphocytic Leukemia	C420, C421, C424	9823
**Myeloid and Monocytic Leukemia**		
Acute Myeloid Leukemia		9840, 9861, 9865–9867, 9869, 9871–9874, 9877–9879, 9895–9897, 9898, 9910–9912, 9920 9891
Chronic Myeloid Leukemia		9863, 9875–9876, 9945–9946
Other leukemias		9860, 9930<br>9801, 9805–9809, 9931<br>9733, 9742, 9800, 9831, 9870, 9948, 9963–9964<br>9820, 9832–9834, 9940
	C420, C421, C424	9827

Table S2. Database sources.

Database Name	Information Extracted
Ontario Cancer Registry (OCR)	Population-based cancer registry data
Canadian Institute for Health Information—Discharge Abstract Database (CIHI-DAD)	Patient-level information on inpatient hospitalization
Activity Level Reporting (ALR)	Patient-level information on radiation, systemic therapy
Ontario Health Insurance Plan Claims Database	Patient-level information on physician claims

New Drug Funding Program (NDFP)	Patient-level cost data on new and expensive hospital-based injectable cancer drugs
Ontario Drug Benefit (ODB) claims database	Patient-level information on all outpatient prescriptions dispensed under the ODB programme
National Ambulatory Care Reporting System (NACRS)	Patient-level information on cost for emergency department visits, cancer clinic visits, and ambulatory care
Ontario Case Costing Initiative (OCCI)	Patient-level costing information
Continuing Care Reporting System (CCRS)	Patient-level information on stays in complex continuing care facilities
Home Care Database	Patient-level information on non-physician home-based care
Same-day surgery database	Patient-level information on same-day procedures requiring no overnight inpatient stay
National Rehabilitation Reporting System (NRS)	Patient-level information on adult inpatient rehabilitation facilities
Ontario Mental Health Reporting System (OMHRS)	Patient-level information on adult mental health services
GAPP decision support systems database (GAPP)	Physician payment information
Estimated Schedule of Benefits (ESTOB)	Information on price associated with each OHIP feecode and suffix

Table S3. Historic CPI for health and personal care in Canada.

Year	CPI Value
2002	100.0
2003	101.2
2004	102.3
2005	104.3
2006	105.8
2007	107.3
2008	109.2
2009	111.9
2010	115.1
2011	117.6
2012	118.7
2013	118.8
2014	120.0
2015	121.9
2016	123.4
2017	125.8
2018	128.0
2019	128.8
2020	129.6
2021	133.3
2022	139.7
2023	147.6

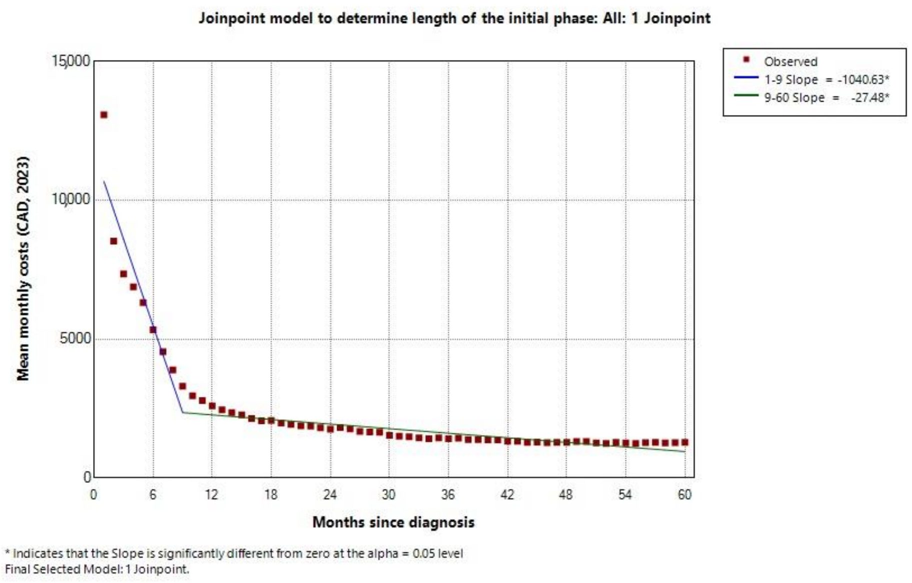

(**a**)

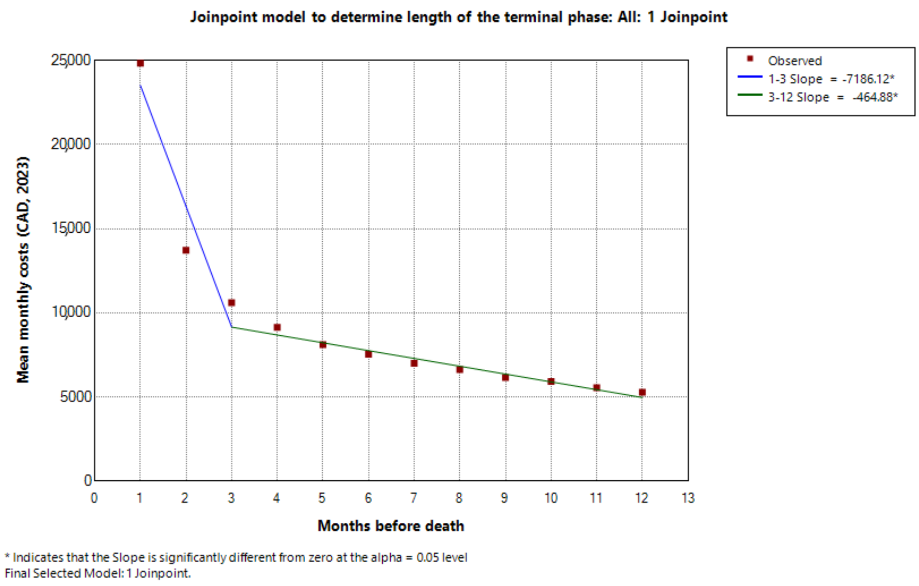

(**b**)

Figure S1. (**a**,**b**): Joinpoint modelling results to determine the duration of (**a**) initial phase and (**b**) terminal phase.

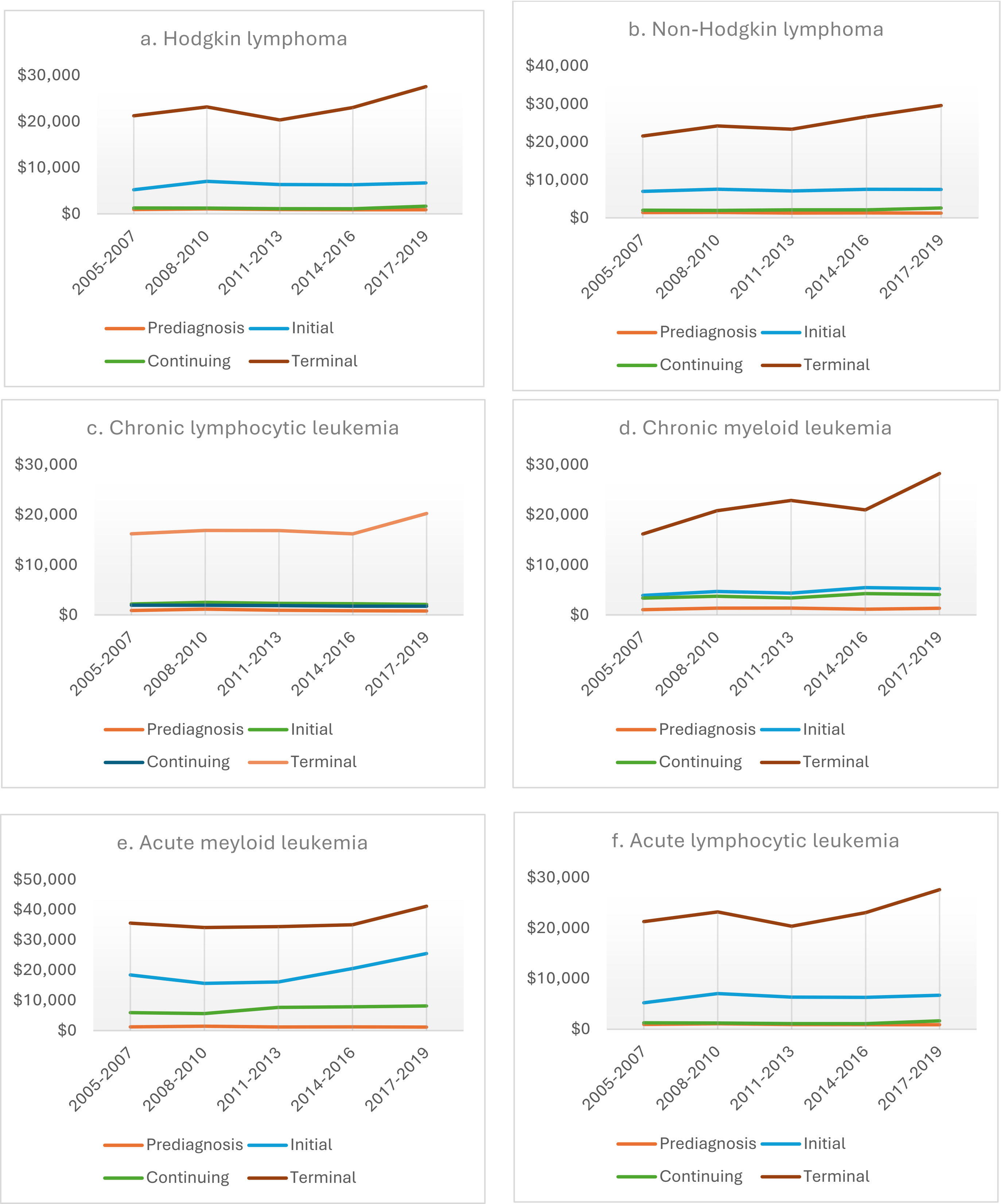
a. Hodgkin lymphoma
$30,000
$20,000
$10,000
$0
2005-2007
2008-2010
2011-2013
2014-2016
2017-2019
Prediagnosis
Initial
Continuing
Terminal
b. Non-Hodgkin lymphoma
$40,000
$30,000
$20,000
$10,000
$0
2005-2007
2008-2010
2011-2013
2014-2016
2017-2019
Prediagnosis
Initial
Continuing
Terminal
c. Chronic lymphocytic leukemia
$30,000
$20,000
$10,000
$0
2005-2007
2008-2010
2011-2013
2014-2016
2017-2019
Prediagnosis
Initial
Continuing
Terminal
d. Chronic myeloid leukemia
$30,000
$20,000
$10,000
$0
2005-2007
2008-2010
2011-2013
2014-2016
2017-2019
Prediagnosis
Initial
Continuing
Terminal
e. Acute meyloid leukemia
$50,000
$40,000
$30,000
$20,000
$10,000
$0
2005-2007
2008-2010
2011-2013
2014-2016
2017-2019
Prediagnosis
Initial
Continuing
Terminal
f. Acute lymphocytic leukemia
$30,000
$20,000
$10,000
$0
2005-2007
2008-2010
2011-2013
2014-2016
2017-2019
Prediagnosis
Initial
Continuing
Terminal

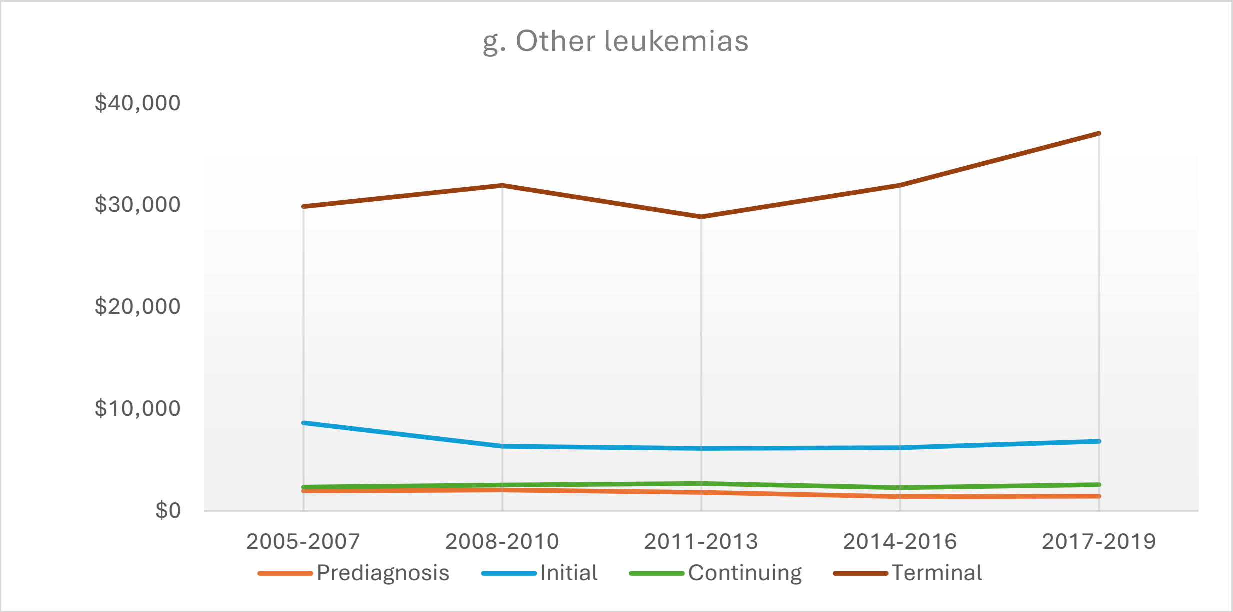

Figure S2. (**a–g**) Trends in phase-specific mean monthly costs grouped by year of diagnosis for subtypes of leukemia and lymphoma (3-year groupings) (CAD 2023).

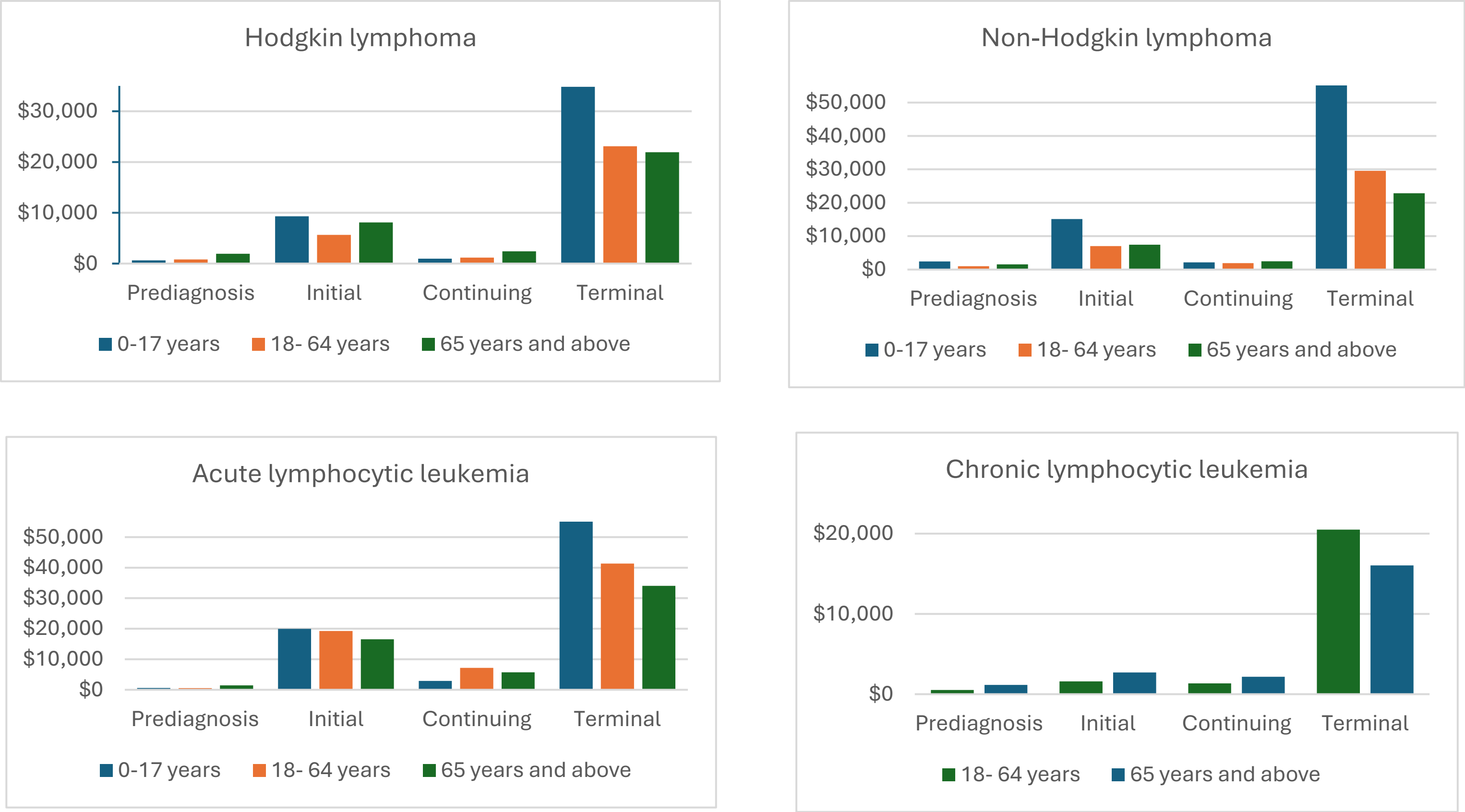

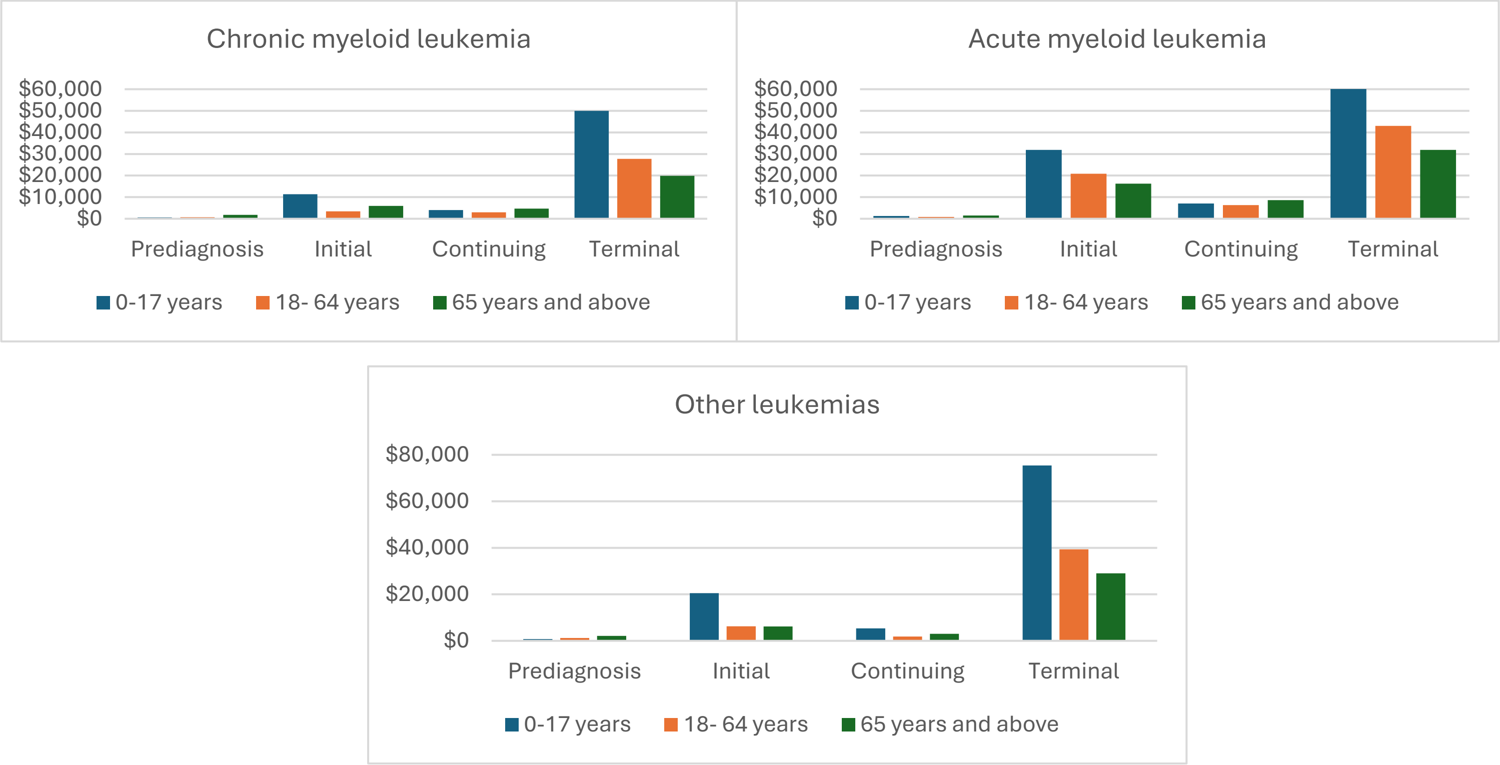

Figure S3. (**a–g**): Phase-specific mean monthly costs grouped by age at diagnosis (CAD, 2023).

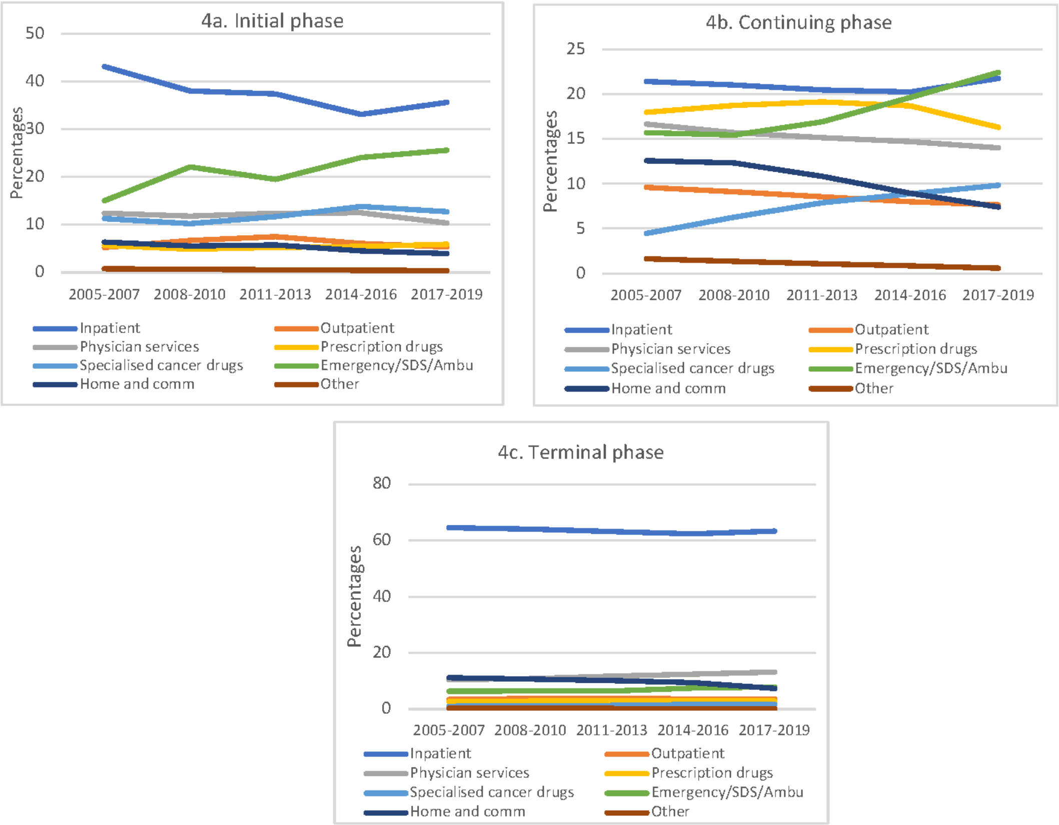

Figure S4. Temporal trends in cost component contribution (% of total costs) for all cancer types combined—(**a**) initial phase, (**b**) continuing phase, (**c**) terminal phase. Note: SDS—same-day surgery; Ambu—ambulatory care; Home and comm—home and community care.

Table S4. (**a–e**): Lifetime costs per patient, stratified by sex and age at diagnosis.

Cancer Subtype	n	Months in Phase			Cost by Phase (CAD 2023)			Lifetime Cost
**A4a. MALES ONLY**								
		Initial	Continuing	Terminal	Initial	Continuing	Terminal	
Hodgkin lymphoma	2825	8.64 (8.58–8.70)	83.75 (81.70–85.79)	2.72 (2.67–2.78)	54,882 (53,061–56,702)	200,218 (180,933–219,502)	16,549 (15,290–17,807)	271,648 (249,285–294,011)
Non-Hodgkin lymphoma	22,980	8.40 (8.38–8.43)	66.19 (65.48–66.89)	2.58 (2.56–2.60)	57,651 (56,781–58,520)	231,923 (225,762–238,085)	41,951 (41,198–42,704)	331,525 (323,740–339,310)
Acute lymphocytic leukemia	1537	8.64 (8.56–8.71)	79.36 (76.47–82.26)	2.54 (2.45–2.64)	157,142 (153,035–161,248)	607,102 (561,034–653,169)	34,165 (31,569–36,761)	798,408 (745,638–851,179)
Chronic lymphocytic leukemia	6578	8.72 (8.69–8.75)	69.51 (68.32–70.71)	2.81 (2.79–2.84)	19,660 (18,576–20,744)	216,340 (208,407–224,274)	31,004 (29,563–32,446)	267,005 (256,545–277,464)
Acute myeloid leukemia	3530	7.38 (7.27–7.49)	45.00 (42.67–47.33)	2.28 (2.25–2.32)	98,528 (95,802–101,254)	308,122 (289,350–326,895)	67,209 (65,117–69,300)	473,859 (450,270–497,449)
Chronic myeloid leukemia	1961	8.30 (8.21–8.40)	60.87 (58.40–63.34)	2.69 (2.64–2.74)	37,797 (35,297–40,298)	373,510 (347,390–399,631)	37,285 (35,075–39,496)	448,593 (417,762–479,424)
Other leukemia	1925	8.34 (8.24–8.44)	66.32 (63.62–69.02)	2.14 (2.06–2.21)	43,844 (40,312–47,377)	217,251 (193,096–241,407)	42,348 (39,744–44,952)	303,444 (273,151–333,736)
**A4b. FEMALES ONLY**								
**Cancer Subtype**	**n**	**Months in Phase**			**Cost by Phase (CAD 2023)**			**Lifetime Cost**
		Initial	Continuing	Terminal	Initial	Continuing	Terminal	
Hodgkin lymphoma	2408	8.68 (8.62–8.74)	85.34 (83.11–87.57)	2.72 (2.65–2.79)	52,588 (51,095–54,081)	196,961 (178,570–215,352)	14,404 (13,214–15,594)	263,954 (242,880–285,027)
Non-Hodgkin lymphoma	19,042	8.44 (8.41–8.47)	69.17 (68.38–69.95)	2.55 (2.53–2.57)	52,305 (51,428–53,182)	219,265 (212,680–225,850)	37,937 (37,143–38,731)	309,507 (301,251–317,762)
Acute lymphocytic leukemia	1157	8.56 (8.46–8.66)	81.03 (77.58–84.47)	2.47 (2.36–2.57)	157,181 (152,066–162,295)	563,087 (503,094–623,079)	32,444 (30,066–34,822)	752,711 (685,226–820,196)
Chronic lymphocytic leukemia	4252	8.76 (8.72–8.80)	74.37 (72.86–75.88)	2.78 (2.74–2.81)	18,167 (17,082–19,252)	210,669 (201,752–219,587)	25,995 (24,665–27,325)	254,832 (243,499–266,164)
Acute myeloid leukemia	3023	7.30 (7.17–7.42)	49.60 (47.03–52.17)	2.24 (2.20–2.29)	99,009 (96,087–101,931)	320,435 (297,461–343,409)	62,928 (60,794–65,062)	482,372 (454,342–510,403)
Chronic myeloid leukemia	1444	8.52 (8.43–8.61)	68.39 (65.50–71.29)	2.68 (2.62–2.74)	36,626 (34,393–38,858)	364,607 (338,697–390,517)	34,019 (31,097–36,940)	435,252 (404,188–466,316)
Other leukemia	1439	8.21 (8.08–8.34)	64.03 (60.82–67.24)	1.92 (1.83–2.00)	39,227 (36,200–42,254)	195,211 (173,815–216,607)	44,025 (41,407–46,644)	278,463 (251,422–305,504)
**A4c. UNDER 18 YEARS**								

Cancer Subtype	n	Months in Phase			Cost by Phase (CAD 2023)			Lifetime Cost
		Initial	Continuing	Terminal	Initial	Continuing	Terminal	
Hodgkin lymphoma	533	8.93 (8.86–8.99)	95.00 (90.57–99.44)	3.00 (3.00–3.00)	83,750 (80,308–87,192)	175,203 (133,144–217,261)	3910 (2408–5412)	262,862 (215,859–309,865)
Non-Hodgkin lymphoma	499	8.72 (8.60–8.83)	86.99 (82.22–91.76)	2.63 (2.40–2.86)	130,511 (121,583–139,439)	360,607 (273,018–448,197)	19,828 (15,095–24,561)	510,946 (409,696–612,196)
Acute lymphocytic leukemia	1627	8.84 (8.78–8.89)	88.80 (86.19–91.42)	2.77 (2.65–2.89)	176,209 (171,895–180,523)	508,038 (471,414–544,661)	14,018 (12,162–15,874)	698,264 (655,471–741,058)
Acute myeloid leukemia	294	8.29 (8.06–8.52)	77.69 (70.67–84.71)	2.77 (2.61–2.93)	257,991 (243,321–272,662)	919,748 (706,553–1,132,944)	56,518 (48,539–64,497)	1,234,258 (998,413–1,470,103)
Chronic myeloid leukemia	52	8.61 (8.11–9.10)	69.49 (56.02–82.97)	3.00 (3.00–3.00)	101,907 (67,402–136,412)	550,573 (316,590–784,556)	32,293 (18,046–46,540)	684,773 (402,038–967,508)
Other leukemia	100	8.34 (7.91–8.76)	98.65 (85.81–111.49)	2.27 (1.73–2.82)	163,178 (130,221–196,136)	780,896 (290,622–1,271,169)	38,782 (28,007–49,558)	982,856 (448,850–1,516,863)

A4d. 18–64 YEARS

Cancer Subtype	n	Months in Phase			Cost by Phase (CAD 2023)			Lifetime Cost
		Initial	Continuing	Terminal	Initial	Continuing	Terminal	
Hodgkin lymphoma	3919	8.76 (8.72–8.80)	86.09 (84.38–87.81)	2.85 (2.80–2.90)	49,749 (48,416–51,082)	196,718 (180,851–212,584)	11,306 (10,323–12,288)	257,772 (239,590–275,955)
Non-Hodgkin lymphoma	18,968	8.61 (8.59–8.63)	77.72 (76.95–78.5)	2.72 (2.70–2.74)	58,370 (57,399–59,342)	265,976 (256,198–275,755)	31,263 (30,464–32,062)	355,610 (344,060–367,159)
Acute lymphocytic leukemia	798	8.40 (8.27–8.53)	63.74 (59.59–67.88)	2.68 (2.59–2.76)	150,027 (144,207–155,847)	741,545 (663,819–819,271)	62,800 (58,744–66,855)	954,372 (866,771–1,041,974)
Chronic lymphocytic leukemia	4058	8.87 (8.84–8.90)	83.34 (81.78–84.91)	2.92 (2.89–2.95)	14,146 (12,979–15,313)	210,418 (200,669–220,166)	18,914 (17,418–20,409)	243,478 (231,067–255,888)
Acute myeloid leukemia	2650	7.93 (7.84–8.03)	55.93 (53.5–58.37)	2.57 (2.52–2.61)	143,355 (139,644–147,067)	471,177 (438,215–504,140)	73,642 (70,645–76,639)	688,174 (648,503–727,845)
Chronic myeloid leukemia	1584	8.66 (8.58–8.74)	78.70 (76.01–81.39)	2.83 (2.76–2.90)	30,007 (28,048–31,967)	453,424 (412,336–494,512)	20,827 (18,494–23,159)	504,258 (458,878–549,638)
Other leukemia	1272	8.70 (8.63–8.78)	75.59 (72.53–78.65)	2.52 (2.42–2.62)	50,550 (46,488–54,612)	247,181 (219,074–275,288)	35,157 (31,479–38,835)	332,888 (297,040–368,735)

A4e. 65+ YEARS

Cancer Subtype	n	Months in Phase			Cost by Phase (CAD 2023)			LIFETIME COST
		Initial	Continuing	Terminal	Initial	Continuing	Terminal	

Hodgkin lymphoma	781	7.84 (7.65–8.03)	61.99 (57.98–66.00)	2.60 (2.53–2.67)	52,606 (49,377–55,836)	169,819 (146,150–193,488)	46,337 (42,886–49,789)	268,763 (238,413–299,113)
Non-Hodgkin lymphoma	22,555	8.23 (8.20–8.26)	56.44 (55.77–57.11)	2.51 (2.50–2.53)	50,965 (50,177–51,753)	176,122 (172,571–179,673)	50,132 (49,340–50,925)	277,219 (272,087–282,351)
Acute lymphocytic leukemia	269	7.25 (6.79–7.7)	49.23 (40.07–58.40)	2.11 (1.97–2.26)	68,963 (61,706–76,220)	176,575 (147,096–206,055)	67,410 (60,802–74,018)	312,948 (269,603–356,294)
Chronic lymphocytic leukemia	6771	8.65 (8.61–8.69)	63.51 (62.39–64.64)	2.78 (2.75–2.80)	21,916 (20,884–22,947)	200,313 (193,480–207,147)	36,098 (34,686–37,509)	258,327 (249,050–267,603)
Acute myeloid leukemia	3609	6.55 (6.41–6.69)	26.28 (24.40–28.16)	2.11 (2.07–2.15)	57,351 (55,365–59,338)	115,676 (108,929–122,424)	62,430 (60,745–64,114)	235,457 (225,039–245,876)
Chronic myeloid leukemia	1769	8.12 (8.01–8.23)	47.00 (44.65–49.35)	2.65 (2.61–2.70)	41,272 (38,758–43,786)	239,275 (225,579–252,971)	49,067 (46,424–51,710)	329,613 (310,761–348,466)
Other leukemia	1192	7.90 (7.76–8.03)	51.64 (49.09–54.19)	1.92 (1.86–1.98)	30,534 (28,030–33,037)	136,251 (125,072–147,430)	51,390 (49,042–53,739)	218,175 (202,144–234,206)
